# G. R. Mines

**Published:** 1914-12

**Authors:** 


### OBITUARY

---

Readers are requested to report promptly the death of all physicians in Western New York, or former residents of this region, or graduates of any medical school in Western New York, and to notify the families of the deceased of our desire to publish adequate obituary notices.

---

**G. R. Mines,** Professor of Physiology at McGill University, Montreal, died November 7. He was a Master of Arts of Sidney Sussex College, Cambridge, 1911, and came to McGill last summer. He was 29 years of age. He had devoted special attention to the physiology of the circulatory and repiratory apparatus. In spite of the short time during which he had been connected with McGill, he had won both the esteem and the affection of his colleagues and students.

